# Global longitudinal strain correlates to systemic right ventricular function

**DOI:** 10.1186/s12947-020-0186-7

**Published:** 2020-01-27

**Authors:** Daniel Samarai, Sandra Lindstedt Ingemansson, Ronny Gustafsson, Ulf Thilén, Joanna Hlebowicz

**Affiliations:** 1 Department of Clinical Sciences, Skåne University Hospital, Lund University, SE-221 85 Lund, Sweden; 2 Department of Cardiology, Skåne University Hospital, Lund University, SE-221 85 Lund, Sweden; 3 Department of Cardiothoracic Surgery, Skåne University Hospital, Lund University, SE-221 85 Lund, Sweden

**Keywords:** Systemic right ventricle, Echocardiography, Atrial switch, Global longitudinal strain

## Abstract

**Abstract:**

**Background:**

The aim of this retrospective study was to evaluate the relationship between right ventricular function derived from cardiac magnetic resonance imaging (CMR), echocardiography and exercise stress test performance, NT-proBNP (N-terminal proB-type natriuretic peptide) level and NYHA class in patients with a systemic right ventricle.

**Methods:**

All patients with congenitally corrected transposition of the great arteries (ccTGA), or transposition of the great arteries after Mustard or Senning procedures, (TGA) followed at our centre who had undergone CMR, echocardiography, an exercise stress test and blood sampling, were included in the study.

**Results:**

We examined 11 patients (six after the Senning procedure, one after the Mustard procedure, and four ccTGA) who have a median age of 32 years (22-67 years). A significant correlation was observed between the systemic ventricular function, expressed as the CMR-derived right ventricular ejection fraction and the right ventricular global longitudinal strain (r= -0.627; p=0.039).

**Conclusion:**

We have demonstrated that in patients with ccTGA or TGA right ventricular global longitudinal strain may be useful in the evaluation of the systemic right ventricular function.

## Introduction

Before the arterial switch operation was introduced in 1975, patients with transposition of the great arteries (TGA) were treated with the Mustard or Senning procedure, also referred to as atrial switch operation [1]. Lifelong annual follow-up is indicated due to the risk of serious complications, such as right ventricular failure, tricuspid valve regurgitation, arrhythmia and sudden death [2]. In congenitally corrected transposition of the great arteries (ccTGA) and after the Mustard or Senning procedure, the morphological right ventricle is the systemic ventricle. The development of systemic right ventricular failure with increasing age is very common in patients after ccTGA, and it has been reported that as many as two thirds of patients with ccTGA suffer heart failure by the age of 45 years [3].

Assessment of the systemic right ventricular function by echocardiography is sometimes complicated, and cardiac magnetic resonance imaging (CMR) is the optimal method. However, it is not possible to use magnetic resonance imaging to examine patients who have a pacemaker, or who suffer from claustrophobia. CMR is also expensive, and availability may be limited. Assessment of the systemic right ventricular function using alternative methods is therefore of interest.

Two-dimensional speckle tracking echocardiography has been used to assess systemic right ventricle function, although experience is limited. Previous studies have shown correlations between systemic right ventricular function and right ventricular global longitudinal strain (GLS), where GLS predicted adverse clinical outcome in patients with TGA [4–6] and ccTGA [5]. In patients with TGA septal longitudinal and circumferential stain have been demonstrated to be reduced [7]. N-terminal proB-type natriuretic peptide (NT-proBNP) levels have also been found to correlate with right ventricular function measured with CMR in patients with TGA [8]. Cardiopulmonary exercise and lung function tests have been recommended to provide additional diagnostic information in the follow-up of patients with congenital heart disease [9]. However, it remains unclear whether right ventricular function measured with CMR and GLS measured with echocardiography are correlated with clinical signs or subjective symptoms of systemic right ventricular dysfunction. The purpose of this retrospective study was therefore to assess the relationship between the systemic right ventricular function derived from CMR, echocardiography, exercise stress test performance, NT-proBNP level, and NYHA class in patients with a systemic right ventricle.

## Methods

The records of all patients with TGA or ccTGA at Skåne University Hospital were obtained from a national registry, “SWEDish registry on CONgenital heart disease” (SWEDCON), which contains information on patients with congenital heart defects and who have undergone heart surgery. The study was approved by the Ethics Review Board in Lund. The selection criteria were: 1) patients with cardiac MRI data, 2) data from other parameters to be collected within the time span of one year, 3) adequate echocardiographic image quality, and 4) patients had reached their teens at time of data collection. Data on NT-proBNP level (ng/L), NYHA class (I-IV), exercise test performance, and the results of CMR and transthoracic echocardiographic loop examination were collected from the patients’ medical records and the SWEDCON database.

All laboratory analysis was carried out at the laboratory of the University Hospital in Lund, using the commercial version of the NT-proBNP ElectroChemiLuminiscence Immunoassay (ECLIA). The lowest detection limit for NT-proBNP was 5 ng/L and the highest detection limit 35 000 ng/L. The coefficient of variability was 5% at 177 ng/L, and 4% at 2100 ng/L.

The parameters measured with echocardiographic loops were: 1) global longitudinal strain (GLS) (%), 2) tricuspid annular plane systolic excursion (TAPSE) (mm), 3) fractional area change (FAC) (%), 4) apical 4 chamber strain (AP4) (%) and 5) short-axis transection strain (SAX) (%). Echocardiographic loop analysis was performed by one cardiologist with experience in echocardiographic examination of grown-ups with congenital heart disease. The venous left ventricular function (LVEF) was measured with echocardiography. The systemic right ventricular function (RVEF) and venous LVEF were derived using CMR. The RVEF (%) was used for assessment of the ventricular function. All patients were examined with CMR in Lund at rest in supine position and during end-expiratory breath hold. A 1.5 T CMR scanner was used for all studies (Philips Achieva, Best, The Netherlands). Images were acquired using sequences with identical temporal and spatial resolution at both field strengths [10–13]. All measurements were done using Segment 1.9 software (http://segment.heiberg.se) [14].

Performance during the exercise stress test was expressed as maximum heart rate (beats per minute, bpm), maximum working capacity (W) and expected maximum working capacity (%). Exercise testing was performed Monark 939E cycle ergometer and Oxygen Pro (Jaeger, Hochberg, Germany). Blood pressure and a 12 lead ECG were monitored during exercise. Continuous data are presented as the median and range. NT-proBNP were not normally distributed and therefore log transformed. The relationship between the systemic RVEF derived from CMR, and exercise test results, logNT-proBNP, and echocardiographic parameters (GLS, TAPSE, FAC, AP4 and SAX) was evaluated using the Pearson correlation coefficient. The relationship between NYHA class and the systemic RVEF, exercise test results, logNT-proBNP, and echocardiographic parameters (GLS, TAPSE, FAC, AP4 and SAX) was evaluated using the Spearman correlation coefficient. The statistical significance was set at p < 0.05. Analysis was performed using IBM SPSS Version 24.

## Results

A total of 11 patients were eligible for inclusion: seven who had undergone atrial switch operation and four with ccTGA. The patient characteristics and measured parameters are given in Table 1. One of the atrial switch operations was the Mustard procedure and the other six were Senning procedures. None of the ccTGA had undergone previous surgery. Moderate tricuspid regurgitation was seen in two patients after atrial switch operation. Severe tricuspid regurgitation or VSD-closure was not observed. Baffle leak was present in one patient, however, hemodynamically insignificant.

**Table 1:** Patient characteristics and descriptive statistics

	After atrial switch operation	ccTGA	Total
Patients (n)	7	4	11
Male/Female (n)	7/0	2/2	9/2
Age (years)	30 (13-37)	35 (25-67)	32 (13-67)
History of clinical arrhythmia (%)	86	50	73
Moderate TR (n)	2	0	1
VSD-closure (n)	0	0	0
NYHA class (n)			
I	5	2	7
II	2	1	3
III	0	0	0
IV	0	0	0
CMR-RVEF (%)	44 (32-60)	48 (23- 59)	44(23 – 60)
Echocardiographic parameters			
GLS (%)	-12.4 (-19.1- -11.0)	-14.6 (-23.8- -7.8)	-13.6 (-23.8 - -7.8)
TAPSE (mm)	17.0 (12.5-18.0)	15.6 (14.3- 16.9)	16.9 (12.5- 18.0)
AP4 (%)	14.3 (11.3-18.3)	15.5 (7.8-19.3)	14.6 (7.8-19.3)
FAC (%)	0.22 (0.18- 0.37)	0.24 (0.21-0.28)	0.22 (0.18 – 0.37)
SAX (%) LVEF (%)	8.0 (3.5-15.8) 41 (34-47)	19.8 40 (27-40)	11.0 (3.5- 19.8) 40.5 (27-47)
NT-pro BNP (ng/L)	231 (75-1349)	1591 (250-3537)	382 (75-2537)
Exercise stress test			
Max. heart rate (bpm)	162 (109-176)	179 (163-184)	170 (109-184)
Max. SBP (mmHg)	170 (125-195)	170 (120-170)	170 (120-195)
Max. working capacity (W)	170 (105-225)	210 (106-218)	180 (105-225)
Expected max. working capacity (%)	66 (43-89)	87 (86-87)	71 (43-89)

Cardiac magnetic resonance imaging (CMR), echocardiographic parameters, NT-proBNP (N-terminal pro B-type natriuretic peptide) and exercise stress test (medians) in seven patients after atrial switch operation and four ccTGA (congenitally corrected transposition of the great arteries) patients. *TR* tricuspid regurgitation. *SBP* systolic blood pressure. *RVEF* right ventricular ejection fraction. *FAC* fractional area change. *GLS* global longitudinal strain. *TAPSE* tricuspid annular plane systolic excursion. *SAX* short axis transection strain. *AP4* apical 4 chamber strain

The median age of the patients with ccTGA was 35 years (25-67 years), and that of the patients undergoing atrial switch operation was 30 years (13-37 years) (Table 1). Seven patients were classified as NYHA class I, three as class II and *none* in the other classes. The venous LVEF derived from echocardiography was 41 % (34-47%) in patients who had undergone atrial switch operation and 40% (27-40%) in ccTGA patients. The RVEF derived from CMR was 44% (32-60%) in patients who had undergone atrial switch operation and 48% (23-60%) in ccTGA patients (Table 1). The median level of NT-proBNP was 382 ng/L (75-2537 ng/L) (Table 1).

**Fig. 1:**
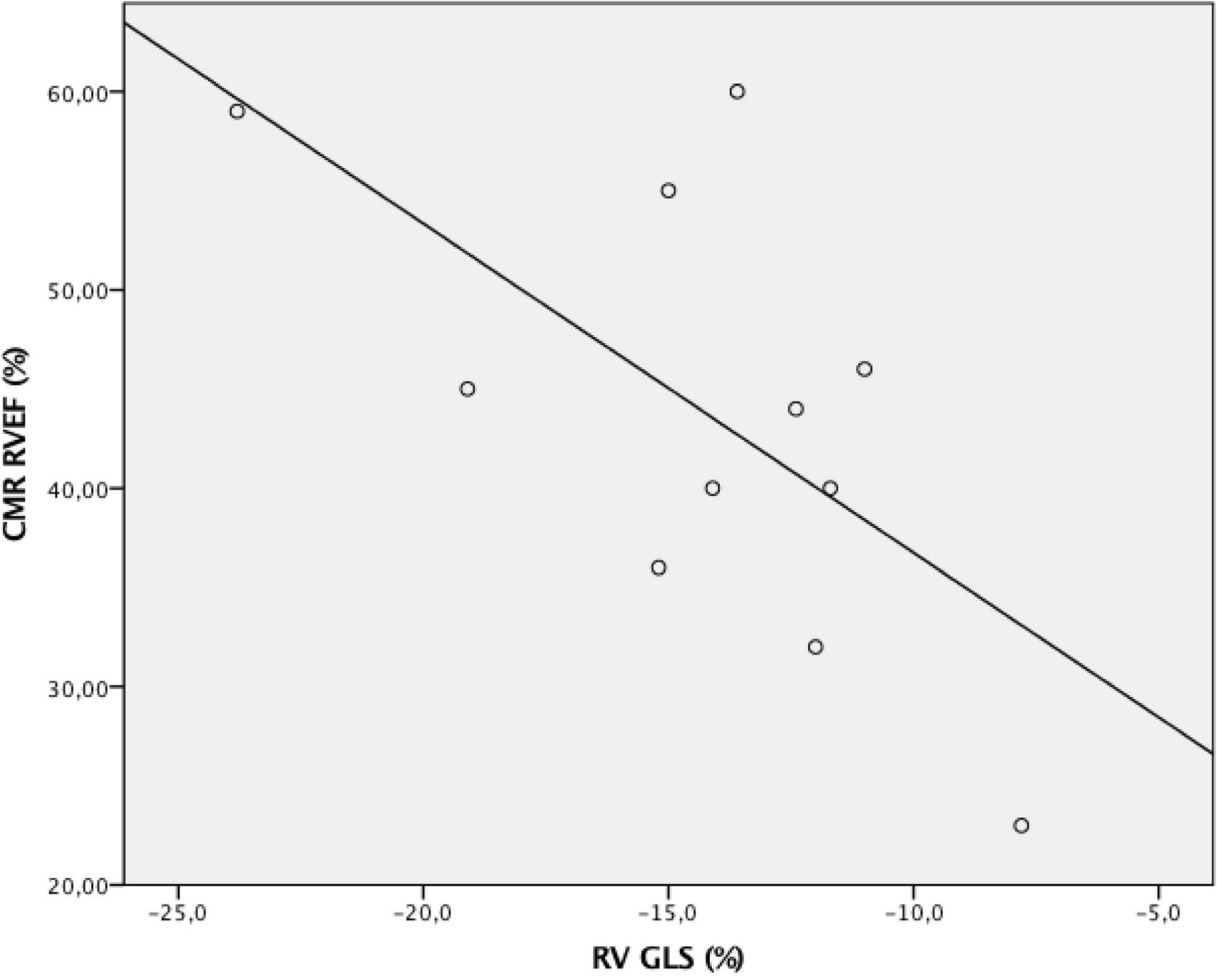
Scatterplot showing the correlation between cardiac magnetic resonance (CMR)-derived systemic RVEF and systemic right ventricular global longitudinal strain (RV GLS)

Table 2 gives the correlation coefficients and p-values. A statistically significant correlation was found between CMR-derived RVEF and right ventricular GLS (r= -0.627; p=0.039) (Fig. 1). No correlation was found between the CMR-derived RVEF and any of the other echocardiographic parameters. -The median maximum working capacity in patients after atrial switch operation was 170 W (105-225W) and in the ccTGA patients 210 W (106-218W). No correlations were found between the CMR-derived RVEF and the exercise test parameters. No statistically significant correlation was found between CMR-derived RVEF and logNT-proBNP or NYHA class.

**Table 2:** Correlation coefficients for CMR-derived right ventricular ejection fraction (RVEF) and given parameters

Parameters	RVEF	
	r-value	p-value
Max. heart rate	0.14	0.70
Max. SBP	0.26	0.48
Max. working capacity	0.60	0.07
Expected max. working capacity	0.47	0.21
logNT-proBNP	-0.615	0.078
NYHA class	-0.114	0.754
GLS	-0.63	0.04
TAPSE	-0.11	0.86
AP4	0.56	0.08
FAC	-0.11	0.74
SAX	0.60	0.27

*SBP* systolic blood pressure. *FAC* fractional area change. *GLS* global longitudinal strain. *TAPSE* tricuspid annular plane systolic excursion. *SAX* short-axis transection strain. *AP4* apical 4 chamber strain

## Discussion

The findings of this study show that the right ventricular GLS were correlated with systemic right ventricular function in patients with a systemic right ventricle. These findings support the use of these methods for the assessment and evaluation of systemic right ventricular function in patients who have undergone atrial switch operation and those with ccTGA. As strain reflects the intrinsic myocardial contractility, in contrast to the ejection fraction, it may be a better method for the assessment of the systemic right ventricle. The aim of this retrospective study was to assess the relationship between the systemic right ventricular function derived from CMR, echocardiography, exercise stress test performance, NT-proBNP level, and NYHA class in patients with a systemic right ventricle. No other has analysed CMR-derived RVEF, echocardiographic non-volumetric measurements, NT-proBNP levels, NYHA class and performance in an exercise test in the same study.

A correlation between CMR-derived right ventricular function and right ventricular GLS has been described previously by Lipczynska et al [4]. In their prospective study of 40 patients with TGA after the atrial switch procedure (mean age 26±5 years), they found a significant linear correlation between the systemic RVEF and GLS, and also between the systemic RVEF and the FAC of the systemic right ventricle. In the mentioned study, systemic right ventricular GLS was proposed to be *able to* discriminate between systemic RVEF *below or above 45%* derived from CMR. Our finding is thus in line with that reported by Lipczynska et al., although weaker (r= -0.627). In contrast to the findings of Lipczynska et al., we did not find a significant correlation between systemic right ventricular systolic function and the right ventricular FAC. This could be due to our smaller study population. It has been suggested that systemic right ventricular GLS could be used instead of right ventricular FAC in patients with systemic right ventricular dysfunction as it has been found to have a higher predictive value, better measurement-remeasurement reproducibility, and can predict adverse clinical outcomes as morbidity and mortality [4]. A reduced septal longitudinal and circumferential strain may contribute to reduced septal work and failure of the systemic right ventricle in TGA patients [7].

The relationship between right ventricular function and NT-proBNP has been reported in previous studies. Plymen et al. reported a statistically significant correlation between CMR-derived RVEF and NT-proBNP in patients after atrial switch operation (35 patients, 54% after Mustard procedure) [8]. Kotaska et al. also reported a relationship between NT-proBNP and the systemic right ventricular systolic function assessed by transthoracic echocardiography in adult patients after the Mustard and Senning procedure [15]. In a systemic review by Eindhoven et al. [16] a significant correlation between BNP and RV function derived by CMR or echocardiography was reported in five of eight studies. Our study joins the studies without reports of a significant relationship between logNT-proBNP and the CMR-derived RVEF. In two of the studies of the mentioned systematic review, a correlation was also seen between the severity of tricuspid regurgitation and BNP. Thus, a possible explanatory factor for our findings, may be the absence of tricuspid regurgitation in our study group and the non-normal distribution of NT-proBNP.

No significant relationship was found between the CMR-derived RVEF and any of the exercise test parameters. Reduced exercise capacity in patients after atrial switch operation has been reported in several studies [17–19]. Previous studies have reported conflicting findings on the correlation between systemic right ventricular function, described by CMR and echoccardiography-derived RVEF and echocardiographic parameters such as GLS, FAC, TAPSE, and exercise capacity [20–23]. In a study on 105 patients with a systemic right ventricle, including both ccTGA and patients after atrial switch operation, Helsen et al. found that neither CMR- nor echocardiographically-derived systolic parameters were correlated to reduced exercise capacity [24]. Although based on a significantly smaller study population, our findings are in line with those of the above-mentioned study, and it is questionable whether a larger study population would be of value, with regard to incoherent findings of earlier studies [20–24]. In the present study, no correlation was found between RVEF and NYHA class. This may have been due to the small variation in NYHA-classes in our study group. However, Plymen et al. found no such correlation either [8]. The absence of correlation between reduced systemic ventricular function and symptoms or exercise capacity, which mainly is seen in acquired cardiac disease, is notable.

This study is limited by the small study population. Also, the time between the dates of examination varied.

## Conclusion

This study has shown that right ventricular GLS measured with echocardiography may be useful in the evaluation of systemic right ventricular function.

## Data Availability

Please contact author for data requests.

## Abbreviations

CCTGA: Congenitally corrected transposition of the great arteries
CMR: Cardiac magnetic resonance imaging
FAC: Fractional area change
GLS: Global longitudinal strain
NT-proBNP: N-terminal proB-type natriuretic peptide
NYHA: New York Heart Association
RVEF: Right ventricular ejection function
SAX: Short-axis transection strain
TAPSE: Tricuspid annular plane systolic excursion
TGA: Transposition of the great arteries

## References

[1] Warnes CA (2006). Transposition of the great arteries. Circulation.

[2] Gelatt M, Hamilton RM, McCrindle BW, Connelly M, Davis A, Harris L (1997). Arrhythmia and mortality after the Mustard procedure: a 30-year single-center experience. J Am Coll Cardiol.

[3] Graham TP, Bernard YD, Mellen BG, Celermajer D, Baumgartner H, Cetta F (2000). Long-term outcome in congenitally corrected transposition of the great arteries: a multi-institutional study. J Am Coll Cardiol.

[4] Lipczynska M, Szymanski P, Kumor M, Klisiewicz A, Mazurkiewicz L, Hoffman P (2015). Global longitudinal strain may identify preserved systolic function of the systemic right ventricle. Can J Cardiol.

[5] Diller GP, Radojevic J, Kempny A, Alonso-Gonzalez R, Emmanouil L, Orwat S (2012). Systemic right ventricular longitudinal strain is reduced in adults with transposition of the great arteries, relates to subpulmonary ventricular function, and predicts adverse clinical outcome. Am Heart J.

[6] Chow PC, Liang XC, Cheung EW, Lam WW, Cheung YF (2008). New two-dimensional global longitudinal strain and strain rate imaging for assessment of systemic right ventricular function. Heart.

[7] Storsten P, Eriksen M, Remme EW, Boe E, Erikssen G, Smiseth OA (2018). Dysfunction of the systemic right ventricle after atrial switch: physiological implications of altered septal geometry and load. J Appl Physiol (1985).

[8] Plymen CM, Hughes ML, Picaut N, Panoulas VF, Macdonald ST, Cullen S (2010). The relationship of systemic right ventricular function to ECG parameters and NT-proBNP levels in adults with transposition of the great arteries late after Senning or Mustard surgery. Heart.

[9] Budts W, Roos-Hesselink J, Radle-Hurst T, Eicken A, McDonagh TA, Lambrinou E (2016). Treatment of heart failure in adult congenital heart disease: a position paper of the Working Group of Grown-Up Congenital Heart Disease and the Heart Failure Association of the European Society of Cardiology. Eur Heart J.

[10] Arvidsson PM, Toger J, Carlsson M, Steding-Ehrenborg K, Pedrizzetti G, Heiberg E (2017). Left and right ventricular hemodynamic forces in healthy volunteers and elite athletes assessed with 4D flow magnetic resonance imaging. Am J Physiol Heart Circ Physiol.

[11] Arvidsson PM, Toger J, Heiberg E, Carlsson M, Arheden H (2013). Quantification of left and right atrial kinetic energy using four-dimensional intracardiac magnetic resonance imaging flow measurements. J Appl Physiol (1985).

[12] Carlsson M, Heiberg E, Toger J, Arheden H (2012). Quantification of left and right ventricular kinetic energy using four-dimensional intracardiac magnetic resonance imaging flow measurements. Am J Physiol Heart Circ Physiol.

[13] Steding-Ehrenborg K, Arvidsson PM, Toger J, Rydberg M, Heiberg E, Carlsson M (2016). Determinants of kinetic energy of blood flow in the four-chambered heart in athletes and sedentary controls. Am J Physiol Heart Circ Physiol.

[14] Heiberg E, Sjogren J, Ugander M, Carlsson M, Engblom H, Arheden H (2010). Design and validation of Segment--freely available software for cardiovascular image analysis. BMC Med Imaging.

[15] Kotaska K, Popelova J, Prusa R (2015). NT-proBNP levels and their relationship with systemic ventricular impairment in adult patients with transposition of the great arteries long after Mustard or Senning procedure. Clin Chem Lab Med.

[16] Eindhoven JA, van den Bosch AE, Jansen PR, Boersma E, Roos-Hesselink JW (2012). The usefulness of brain natriuretic peptide in complex congenital heart disease: a systematic review. J Am Coll Cardiol.

[17] Hechter SJ, Webb G, Fredriksen PM, Benson L, Merchant N, Freeman M (2001). Cardiopulmonary exercise performance in adult survivors of the Mustard procedure. Cardiol Young.

[18] Trojnarska O, Gwizdala A, Katarzynski S, Katarzynska A, Oko-Sarnowska Z, Breborowicz P (2010). Evaluation of exercise capacity with cardiopulmonary exercise testing and BNP levels in adult patients with single or systemic right ventricles. Arch Med Sci.

[19] Paul MH, Wessel HU (1999). Exercise studies in patients with transposition of the great arteries after atrial repair operations (Mustard/Senning): a review. Pediatr Cardiol.

[20] Norozi K, Buchhorn R, Alpers V, Arnhold JO, Schoof S, Zoege M (2005). Relation of systemic ventricular function quantified by myocardial performance index (Tei) to cardiopulmonary exercise capacity in adults after Mustard procedure for transposition of the great arteries. Am J Cardiol.

[21] Tutarel O, Orwat S, Radke RM, Westhoff-Bleck M, Vossler C, Schulke C (2016). Assessment of myocardial function using MRI-based feature tracking in adults after atrial repair of transposition of the great arteries: Reference values and clinical utility. Int J Cardiol.

[22] Shafer KM, Mann N, Hehn R, Ubeda Tikkanen A, Valente AM, Geva T (2015). Relationship between Exercise Parameters and Noninvasive Indices of Right Ventricular Function in Patients with Biventricular Circulation and Systemic Right Ventricle. Congenit Heart Dis.

[23] Ladouceur M, Redheuil A, Soulat G, Delclaux C, Azizi M, Patel M (2016). Longitudinal strain of systemic right ventricle correlates with exercise capacity in adult with transposition of the great arteries after atrial switch. Int J Cardiol.

[24] Helsen F, De Meester P, Van De Bruaene A, Gabriels C, Santens B, Claeys M (2018). Right ventricular systolic dysfunction at rest is not related to decreased exercise capacity in patients with a systemic right ventricle. Int J Cardiol.

